# Editors Not Always Responsible

**Published:** 1897-07

**Authors:** 


### EDITORS NOT ALWAYS RESPONSIBLE.

There has recently been some very severe criticisms upon editors of dental journals for permitting certain advertisements upon their pages of a character injurious to professional interests and in direct violation of the code of ethics.

The objections raised to the advertisements in question have been justified by their character, and no reasonable opposition can be made to the resolution adopted by the Academy of Dental Science of Boston and published in this journal. The adoption of this is most satisfactory evidence that at least one society has had a watchful care that nothing shall appear unworthy professional character without due notice. If all dental organizations were equally careful the standard of professional publications would soon be raised beyond criticism.

It, however, must be borne in mind that editors are not responsible for the matter that appears upon advertising pages. This belongs to the business side of journalistic work and with which they have nothing to do.

All well-arranged periodicals must be conducted upon business principles, or they will fail, a fact seemingly forgotten by those who are too apt to view only one side of a question. The editor is quite sufficiently burdened with the preparation of the journal under his charge, and cannot supervise the business portion, even if qualified for that work.

The question of the intermingling of trade with professional journals has been a bone of contention for many years, and, in the view of the writer, has not always been justly treated. The contention made by those who oppose all advertisements has been that these lowered the professional standard and should not be admitted to the pages of any well-conducted journal; that these should be confined strictly to professional matters. This, to our view, is not only unreasonable, but would, if carried out, be productive of great injury to the very profession the critics seek to serve.

In a recent article the writer sought to show that there were two sides to the dental profession, both equally important, and, while distinct, were essential to its true progress. The strictly professional and trade should not be permitted to overlap each other, but can, without detriment to either, work side by side, each a mutual help and each worthy of respect in its own particular line of work. If this view be the correct one, there can be no loss of professional respect to have both sides represented in the same journal. To have this requires care and discrimination as to the matter permitted to appear. This is, at times, a problem requiring a mind trained to view both sides of the question. As this quality is not always attainable, errors of judgment are possible and frequently occur.

There is another view to take of this matter, not always considered, and that is that the advertising pages of a journal are oftentimes, if not always, more valuable than the pages devoted to professional matter. The mass of dentists care more for these than they do for the subjects considered in the periodical. Those who live in large cities and surrounded continuously with the active elements of trade cannot appreciate the position of one practising in a remote section, isolated from all connection with professional thought, rarely or never coming in direct contact with dental exhibits and depending entirely upon the illustrations of advertisers for his knowledge of improvements being made in dental mechanism. The trade side of journalism is to him the open door to progress, enabling him to continue in touch directly and effectively with his colleagues more favorably situated. More than this, these pages become part of the history of the period, growing more and more valuable as time passes on. The evidence of this is to be found in the fact that collectors of dental literature will not permit their periodicals to be bound without the advertisements, and those who have had occasion to look over old journals turn with more interest to the advertisements than they do to the text.

Is it not time, then, that this hue and cry against advertisements should cease, and in its place accept the broader view that progress cannot effectively be made by working entirely in one direction or in condemning the labor of others?

